# Microbial evasion of the complement system: a continuous and evolving story

**DOI:** 10.3389/fimmu.2023.1281096

**Published:** 2024-01-04

**Authors:** Mariam T. Heggi, Hanzada T. Nour El-Din, Dina I. Morsy, Noha I. Abdelaziz, Ahmed S. Attia

**Affiliations:** ^1^ Clinical Pharmacy Undergraduate Program, Faculty of Pharmacy, Cairo University, Cairo, Egypt; ^2^ Department of Microbiology and Immunology, Faculty of Pharmacy, Cairo University, Cairo, Egypt; ^3^ School of Pharmacy, Newgiza University, Giza, Egypt

**Keywords:** complement evasion, serum resistance, pathogens, virulence factors, regulation

## Abstract

The complement system is a fundamental part of the innate immune system that plays a key role in the battle of the human body against invading pathogens. Through its three pathways, represented by the classical, alternative, and lectin pathways, the complement system forms a tightly regulated network of soluble proteins, membrane-expressed receptors, and regulators with versatile protective and killing mechanisms. However, ingenious pathogens have developed strategies over the years to protect themselves from this complex part of the immune system. This review briefly discusses the sequence of the complement activation pathways. Then, we present a comprehensive updated overview of how the major four pathogenic groups, namely, bacteria, viruses, fungi, and parasites, control, modulate, and block the complement attacks at different steps of the complement cascade. We shed more light on the ability of those pathogens to deploy more than one mechanism to tackle the complement system in their path to establish infection within the human host.

## 1 The complement system: introduction

Our planet is heavily populated by microscopic organisms that are trying to evade their hosts in search of a place where they can live and prosper. Thus, the homeostasis of the host is threatened by a vast array of allergenic or toxic substances. Each pathogen has a special mechanism by which it replicates, spreads, and unintendedly alters the host’s functions. It is not surprising, therefore, that our immune system is equipped with a complex array of defensive mechanisms to oppose these alterations. At the same time, our immune system works to avoid responses that produce excessive damage to self-tissues or that might abolish beneficial commensal microbes (1).

The immune system is well-constructed upon two general systems, innate and acquired immunity. Our innate immunity functions through its soluble proteins and bioactive small molecules that are constitutively present in biological fluids, such as the complement proteins, defensins, and ficolins (1). The complement system is a highly sophisticated biological reaction system that primarily augments the opsonization, phagocytosis, and lysis of the target cells. However, it does not merely “complement” – though it upholds the name – but it forms an open border between innate and adaptive immunity, ultimately serving the purpose of maintaining homeostasis and tissue integrity (2).

Multiple functions of the complement system are carried out by effector fragments, which are inactive zymogens that are activated into proteases of 30-50 proteins, most of which are synthesized in the liver. Those proteases cleave other components successively in amplification pathways leading to exponential generation of the final effectors (3).

### 1.1 Complement activation

**Figure 1:**
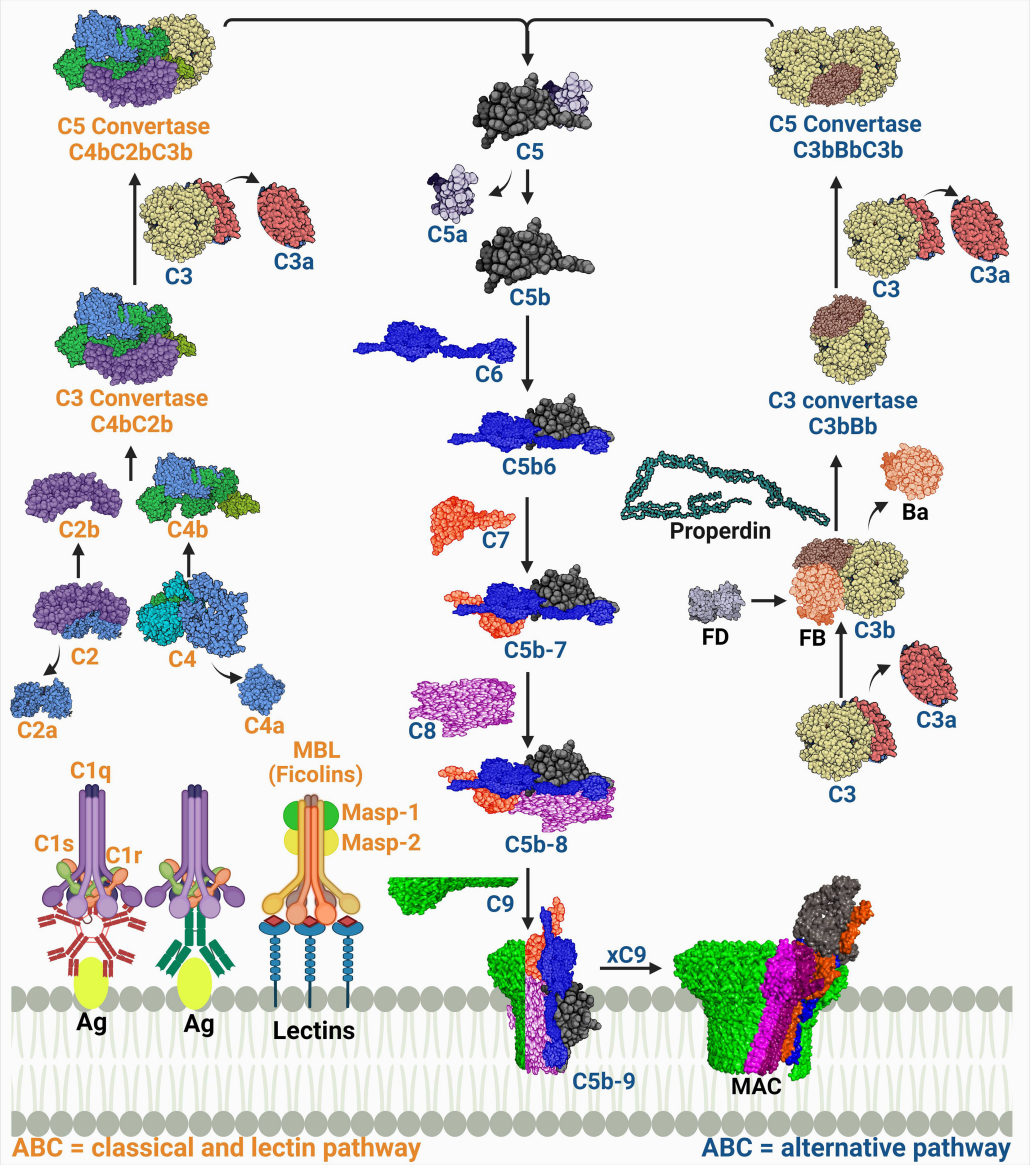
A schematic diagram showing the three complement activation pathways converging at a common terminal route of forming the membrane attack complex (MAC). The labels of the components of the classical and lectin pathways are colored in orange, while those of the alternative pathway are colored in dark blue. The proteins’ codes and structures were retrieved from the Protein Data Bank, while the figure was generated using BioRender.

The complexity of the complement system is not solely in its composition, but it also extends to include its initiation via three pathways (**Figure 1**). The pathways intertwine enough to be called a maze; however, they have distinct features starting from their initiators and the pattern recognition molecules (PRMs) they recognize.

#### 1.1.1 Complement system initiation and generation of the C3 convertase

The PRM for the classical pathway is the C1q. It recognizes immune complexes such as antigen-bound immunoglobulin G (IgG) and IgM, thus this pathway is also known as the ‘antibody-dependent’ pathway. A conformational change then occurs allowing C1q, a hexamic collagen-like protein, to interact with C1r and C1s and form activated C1qr2s2 (4, 5). The activated C1qr2s2 subsequently cleaves C4, then C2 in a Ca^2+^ dependent reaction which forms the C4b and C2b complex (C4bC2b) known as the classical pathway C3 convertase. This complex is responsible for the cleavage of C3 into C3a and C3b. The latter will then be able to induce proteolytic activity and activate the common terminal pathway (6, 7). Other structures such as C-reactive protein (CRP) can also activate C1q in an antibody independent manner (8).

The C1q analog in the lectin pathway is the mannose-binding lectin (MBL). The MBL is the first known molecule to initiate this pathway and it recognizes monosaccharides exposing horizontal 3′- and 4′-OH groups as in mannose, glucose, and N-acetyl glucosamine but only when organized in a pattern fitting array (9). There are other humoral PRMs found in humans that can initiate the lectin pathway as well which are ficolins (M-, L-, and H-ficolin; also called ficolin-1, -2, and -3), and collectin 11 (CL11 or CL-K1) (10). The PRMs interact with serine proteases, known as MBL-associated serine proteases (MASPs), which are initially present as zymogens within the complexes. The complement cascade is initiated when the recognition molecules bind to their target and trigger the proteolytic step which starts with auto activation of MASP-1, which successively cleaves MASP-2. Both MASP-1 and MASP-2 cleave C2; however, C4 is cleaved only by MASP-2. Thus, both enzymes play an essential role in the subsequent assembly of the C4bC2b (CP and LP C3 convertase) (11, 12). The MASP-3 is a competitor for MASP-2 in binding to MBL. Although this competition slows LP activation, MASP-3 is considered the primary activator of the alternative pathway (AP) protease, factor D, in the human body fluids (13, 14). It is important to note that CP and LP are always in an inactive form, and they need PRMs to be activated.

On the contrary, the AP is in a constitutively activated state, referred to as the ‘tick-over’. This occurs through the spontaneous hydrolysis of a highly reactive thioester bond in C3 producing an active C3 form termed C3(H_2_O). This active form is able to form a complex with factor B “C3(H_2_O)B”. This complex is a substrate for factor D, which cleaves the B protein to form a short-lived soluble C3 convertase (C3(H_2_O)Bb). C3 is cleaved to give C3a, an anaphylatoxin (recruiter of inflammatory cells), and C3b that will likewise bind to factor B assembling the C3 convertase (C3bBb) and it then leads to the amplification loop (11, 14). Activated neutrophils release a protein called properdin, which stabilizes the C3bBb by binding to C3b to stop its cleavage by factors H and I. Later it was found that properdin can initiate the convertase assembly by either nonspecific covalent binding to C3b, C3bB, and/or C3bBb or by specific noncovalent binding to the target which as a result, increases the convertase assembly and C3b deposition (15). In addition, the generation of C3d from the C3 cleavage enhances the opsonization process together with iC3b and C3b (16).

All this is manifested by the presence of the C3 cleavage products, C3b and C3(H_2_O), in plasma in the normal physiological state. This low level of activation is the core of the complement surveillance activity. It is proposed that the AP C3 is a ‘contact-activated’ protein, which means that upon interaction with a biological or artificial interface, the C3 hydrolysis rate will be accelerated, ensuring the continuous supply of reactive C3b that can deposit on the surfaces of pathogens (17, 18).

A top view of the three pathways (**Figure 1**) indicates that each of them runs singularly; however, C3 is a common product of all pathways and its cleavage by the respective convertase determines the first complement effector activity. Although the three pathways are not linearly aligned, they represent a coherent network entangled on the same route to give effector functions and produce immune surveillance. The CP and the LP share the same convertase structure (C4bC2b), while the AP convertase is distinct (C3bBb) (19).

#### 1.1.2 Generation of the C5 convertase

The classical/lectin pathway C5 convertase, C4bC2bC3b, and the alternative C5 convertase, C3bBbC3b, are both products of the binding of C3b, which is deposited from the amplification loop to the pre-assembled C3 convertases. Both C3 convertases catalyze the proteolysis of C3 into C3a and C3b and the subsequent cleavage of C5 into C5a and C5b (20). C5 cleavage is mostly managed by the AP, while properdin acts as a stabilizer for the convertase end products. However, C5 cleavage activation is not yet fully understood (21). The C3 convertase complex (C3bBb) switches the specificity of the enzyme substrate affinity from C3 to C5 cleavage into C5a and C5b (22). C5b is momentarily available to bind with C6, hence initiating the terminal pathway.

#### 1.1.3 Assembly of the membrane attack complex

The stable complex (C5b-6) sequentially recruits proteins C7 and C8 and polymers of C9, leading to the multimeric complex assembly of the membrane attack complex (MAC). The complex penetrates the phospholipid bilayer as a result of the structural transition of its components that exposes hydrophobic regions, enabling its binding to the cell membrane (23). In order to induce a functional effect on the lipid bilayer, in which a tubular transmembrane pore is formed, 10 to 18 C9 molecules are needed (24). This pore acts as a channel, diffusing ions and small molecules, and killing the cell by osmotic instability.

However, not all cells are vulnerable to lysis by MAC. For instance, metabolically active nucleated cells can exhibit resistance and repair mechanisms against complement lysis (25). In addition, Gram-positive bacteria are naturally protected from lysis by their thick cell wall, although sub-lytic activity of the MAC can still induce signal transduction pathways (24, 26).

### 1.2 Complement system regulators

The undeniably important role of the complement system makes its regulation of even greater importance. Hence, a balance is maintained to ensure the effective functioning of the complement system and avoid possible autologous harm. This balance is achieved through a group of regulators and inhibitors that also keep the severity and propagation of the complement cascade on track. These regulators are either cytoplasmic or membrane-bound and they act on different and sometimes multiple levels of the cascade according to the pathway involved (27) (**Figure 1**). A better understanding of the role of each of these regulators is a pillar in safeguarding against pathogen evasion of the complement system (28).

#### 1.2.1 Initiation of the C3 convertase and its regulation

The activation of the two closely connected CP and LP pathways is inhibited by the C1 inhibitor (C1INH) - a plasma serine proteinase (SERPIN1) - that irreversibly binds to, and hence inhibits, both the C1r and C1s of the CP and the MASP-1 and MASP-2 of the LP (3). Moreover, the LP is inhibited by the binding affinity of the small mannose-binding-lectin associated protein (sMAP) and MAP-1 to MBL and ficolins, as they are non-proteolytic splice products of the MASP2 and MASP1/3 genes, respectively. They possess no similar activity to MASP-1 or MASP-2 causing the inhibition of the LP (11).

The C3 convertase of the AP is regulated by factor H (FH) and its homolog factor H-Like protein 1 (FHL-1), which can either express decay accelerating activity or aid in the C3b degradation (28, 29). Also, C4 binding protein (C4BP) is a cofactor of FI that accelerates the decay of the CP and LP C3 convertase (C4bC2b), and binds C4b (8).

The membrane bound regulators, such as membrane cofactor protein (MCP or CD46) which has cofactor activity for FI and the decay accelerating factor (DAF or CD55), accelerate the decay of the C3 convertases in the three pathways. Similarly, the complement receptor 1 (CR1 or CD35) expresses decay accelerating activity and functions as a cofactor for FI-mediated cleavage of C3 convertase (3, 30). Finally, the carboxypeptidase-N inactivates the complement anaphylatoxin peptides C3a and C5a (8).

#### 1.2.2 Regulation of the C5 convertase and MAC

Regulation of the complement system extends reaching the inhibitors of the C5 convertase. Those inhibitors include the DAF and FH that have destabilizing, and decay accelerating activity (11). The final effector molecule of the complement, the MAC, is regulated by protectin (CD59), a cell-based protein that inhibits the binding of C9 to the C5b-8 complex and hence prevents the MAC assembly (31). Additionally, soluble plasma based regulators, such as vitronectin (Vn) and clusterin, bind to C5b-7 and prevent the assembly of the MAC (28).

### 1.3 Complement evasion

The complex and crucial roles of the complement system in both adaptive and innate immunity have made it an integral part of the relentless rivalry between the immune system and pathogens (32). This rivalry begins with microbes trying to evade detection by the complement system, which is the very early, and somehow frontline, force of immunity to block further effector functions and reactions. There is a clear distinction between infectious microbes that are successfully recognized and eliminated, and pathogenic microbes that survive in an immunocompetent host (33). The emergence of multiple strategies to evade the complement system, impair its cascade, and prevent its effector functions is no surprise. The evasion of the complement system by a pathogen is now a battle of wits as many strategies targeting different layers of the complement system have unfolded in the past decades (34). There is a repertoire of mechanisms utilized by multiple pathogens that are well-established and understood. Known mechanisms include the recruitment and binding of complement system regulators or expression of regulator-like substances by pathogens, as well as, expression of inhibitors (35). Additionally, the secretion of proteases specifically against target complement proteins is considered a virulence mechanism (36, 37). There are common features that can be seen in the aforementioned mechanisms which are beneficial in exploiting the host defence system. The first feature is the sequence diversity of individual escape proteins (34). Also, the multiplicity of mechanisms that are utilized by a single pathogen represents another elusive approach against the complement system (38). Finally, the redundancy in the timely expression of several proteins directed to complement regulators or host defences is a common feature in multiple pathogens (33).

#### 1.3.1 Influencing the complement activation: the beginning of the fight

**Figure 2:**
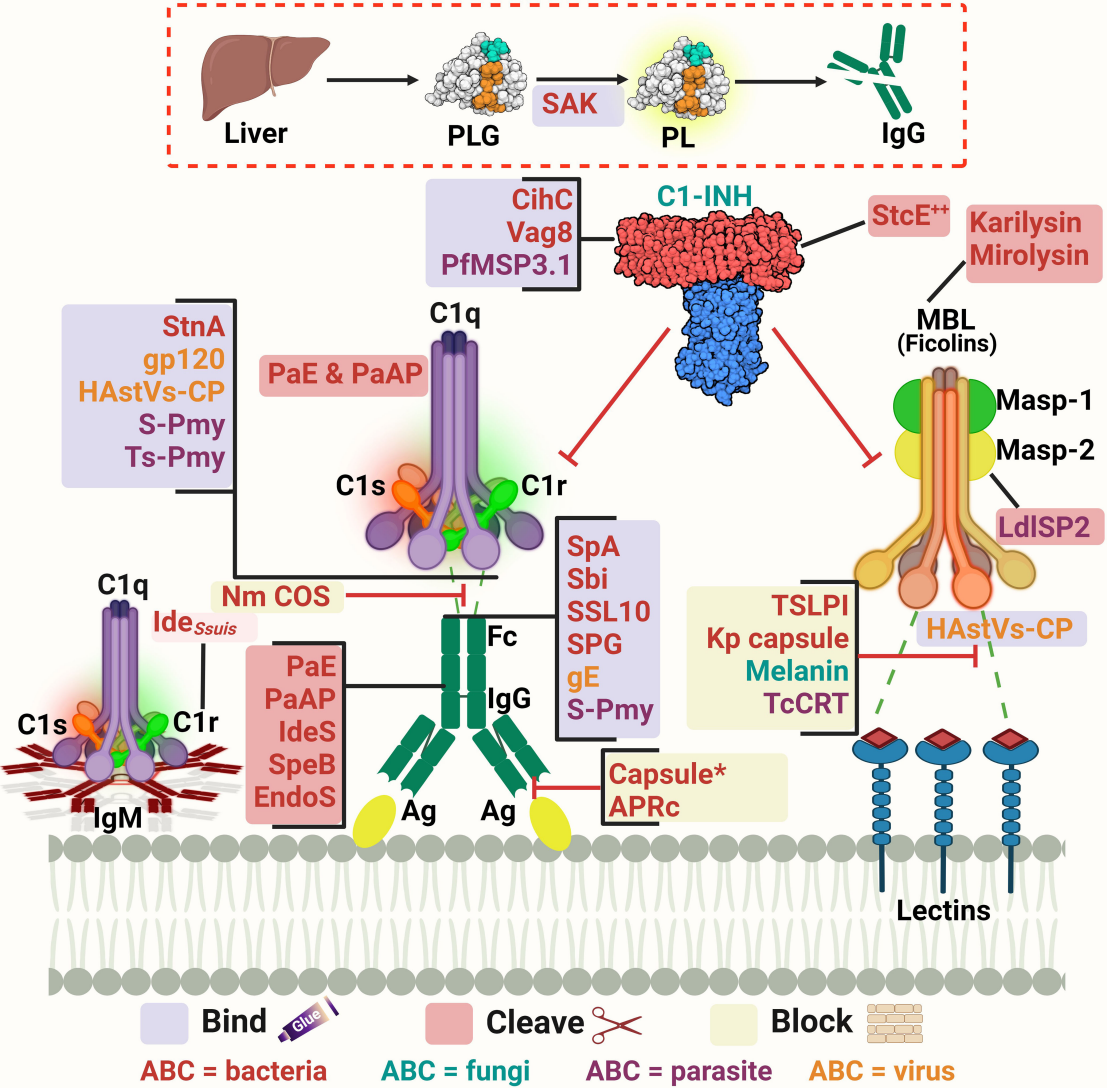
A schematic diagram showing microbial virulence factors involved in targeting the initiation phase of the complement system. The labels for the bacterial factors are shown in red, the fungal ones in turquoise, the parasite-related ones in purple, and finally the viral factors in orange. The mechanism by which those proteins affect the pathway is categorized into binding, shaded in purple; cleavage, which is shaded in dark pink; and blocking, which is highlighted in beige. The proteins’ codes and structures were retrieved from the Protein Data Bank, while the figure was generated using BioRender. The * indicates that the capsules of multiple pathogens perform the same function. The ^++^ indicates that the StcE cleavage of the C1-INH activates it rather than inhibiting it.

As pathogens begin their survival struggle against the immune system, evasion of the complement activation, and effector functions becomes a matter of life or death. Targeting the initiation phase of the complement system is a strategy seen in multiple organisms (33). The microbial factors deployed in these mechanisms are summarized in **Table 1** and **Figure 2**. The CP and LP have PRMs that are possible targets for microorganisms, for example, the Gram-negative bacterium *Pseudomonas aeruginosa* produces two proteases, *Pseudomonas* elastase (PaE) and *Pseudomonas* alkaline protease (PaAP), that cleave immunoglobulins and C1q, hence inhibiting the CP activation (47). Another mechanism is the inhibition of the C1q and binding of immunoglobulin which is seen in *Neisseria meningitidis* by its capsular oligosaccharides that interfere with C1q and IgG binding (46). The *Streptococcus* spp. have multiple evasion mechanisms including cleavage of the IgG by the IgG-protease of *S. pyogenes* (IdeS) and the streptococcal pyrogenic exotoxin B (SpeB), as well as the endoglycosidase (EndoS) (52, 53). Also, the capsular polysaccharides of *S. pneumoniae* decrease complement initiation by minimizing the binding of the complement components (54). Additionally, the streptococcal protein G (SPG) binds to the Fc portion of IgG, inhibiting its interaction with C1q (55). In the emerging zoonotic pathogen *S. suis*, the cell wall anchored heme-binding protein SntA was shown to bind C1q, interfering with the CP activation (56). It has also been suggested that SntA can interfere with both CP and LP through complement component consumption (56). The same pathogen secretes the Ide*_Ssuis_* protease, which is highly specific to porcine IgM and hence, blocks CP activation via this immunoglobulin (57). *Rickettsia* spp. express the APRc, which is an Ig-binding protein that binds in a non-immune way with different immunoglobulins from different hosts, blocking the activation of the CP (48).

**Table 1:** Microbial strategies for influencing the complement activation.

Organism	Microbial factor	Action	Ref
A) Bacteria			
*B. anthracis*	(PDG) capsule	Blocks IgG binding	(39)
*Borrelia* spp.			
i) *B. burgdorferi*	TSLPI	Prevents MBL binding	(40)
ii) *B. recurrentis*	CihC	Binds C1-INH	(41)
*B. pertussis*	Vag8	Binds C1-INH	(42)
*E. coli*	K1 capsule	Masks surface antigens	(43)
	StcE	Cleaves C1-INH leading to its potentiation	(44)
*K. pneumoniae*	Capsule	Camouflages to avoid recognition by LP molecules	(45)
*N. meningitidis*	Capsular oligosaccharides	Interfere and inhibit C1q and IgG	(46)
*P. aeruginosa*	PaE & PaAP	Cleave immunoglobulins and C1q	(47)
*Rickettsia* spp.	APRc	Binds Igs	(48)
*S. aureus*	SpA, Sbi, & SSL10	Bind to IgG Fc ➔↓ interaction with C1q	(49, 50)
	SAK	Binds PLG ➔ activated to plasmin➔ cleaves IgG	(51)
*Streptococcus* spp.			
i) *S. pyogenes*	IdeS, SpeB, & EndoS	Cleavage of IgG	(52, 53)
ii) *S. pneumoniae*	Capsule	↓ the binding of the complement components	(54)
	SPG	Binds to IgG Fc ➔↓ interaction with C1q	(55)
iii) *S. suis*	StnA	Binds C1q	(56)
	Ide*_Ssuis_*	Cleaves IgM	(57)
*S. sonnei*	Capsule	Masks surface antigens	(58)
*T. forsythia*	Karilysin & Mirolysin	Degrade MBL, ficolin-2, and ficolin-3	(59, 60)
*V. cholerae*	Capsule	Masks surface antigens	(61)
B) Fungi			
*P. brasiliensis*	Melanin-like	Masks the cell wall lectin receptor targets	(62)
C) Viruses			
Herpes virus	gE	Binds to IgG Fc ➔↓ interaction with C1q	(63)
HAstvs serotypes 1-4	HAstVs-CP	Interacts with C1q and MBL	(60)
HIV-1	gp120	Binds C1q	(64)
D) Parasites			
*L. donovani*	LdISP2	Inhibits MASP2	(65)
*P. falciparum*	PfMSP3.1	Binds C1-INH	(66)
*Schistosoma* spp.	S-Pmy	Binds C1q and IgG	(67, 68)
*T. spiralis*	Ts-Pmy	Binds C1q	(69)
*T. cruzi*	TcCRT	Inhibits ficolin-2 recognition	(70)

“↓” symbol indicates a meaning of '' decrease'' and “➔” symbolize the meaning of '' leads to".

In the case of *Staphylococcus aureus*, protein A (SpA) binds to the Fc region of IgG, diminishing its interaction with C1q. There is also the staphylococcal binder of immunoglobulins (Sbi) and the staphylococcal superantigen-like protein 10 (SSL10) which inhibit the CP activation in the same way (49, 50). Moreover, *S. aureus* produces distinct proteolytic enzymes such as staphylokinase (SAK) which binds plasminogen (PLG) to its surface, resulting in its activation to plasmin which cleaves bound IgG and blocks the complement activation (51). On another front, the poly-γ-D-glutamate (PDG) capsule of *Bacillus anthracis* protects this pathogen from opsonic phagocytosis by interfering with complement activation and blocking the binding of IgG and C3b (39). The use of capsules to mask microbial surface antigens and evade complement activation and subsequent killing has been seen in other pathogens such as *Escherichia coli* (43), *Shigella sonnei* (58), and *Vibrio cholerae* (61).

As for viruses, the Herpes virus has several glycoproteins that target the complement system. For instance, glycoprotein E (gE) possesses Fc-receptor properties to diminish the CP-essential antibody recognition (63). The human immunodeficiency virus type 1 (HIV-1) targets the C1q as well and binds it with the envelope glycoprotein gp120 (64). While in parasites, *Schistosoma* spp. use the protein paramyosin (S-Pmy) which has binding affinities for both C1q and IgG (67, 68). On the other hand, the homolog of this protein in *Trichinella spiralis* (Ts-Pmy) inhibits the CP through binding to C1q (69). 

This multitude of mechanisms that target the PRMs is not confined to the CP only but is also seen in the LP. In the case of *Leishmania donovani*, its inhibitor of serine peptidases 2 (LdISP2) demonstrated a potent effect on MASP2 of the LP leading to the blockage of the complement activation cascade (65). For instance, some strains of *Klebsiella pneumoniae* resort to camouflaging their capsular composition to avoid recognition by the LP molecules (45). The tick-borne *Borrelia burgdorferi* deviously benefits from the tick salivary lecithin pathway inhibitor protein (TSLPI) at the tick bite site to inhibit LP activation by preventing MBL binding (40). Also, the cleavage of the LP PRMs by proteases is seen in *Tannerella forsythia*, a pathogen strongly associated with periodontitis, that produces two metalloproteinases called karilysin and mirolysin that degrade the MBL ficolin-2 and ficolin-3, hence inhibiting the LP (59, 60). The parasite *Trypanosoma cruzi* specifically targets the LP activation by expressing the surface protein *T. cruzi* calreticulin (TcCRT) to inhibit the ficolin-2 mediated activation of the LP (70). The *Paracoccidioides brasiliensis* yeast masks the cell wall lectin receptor targets with a melanin-like pigment and hence prevents the complement-dependent phagocytosis (62). Finally, the human astroviruses (HAstVs) serotypes 1-4 are able to inhibit the complement system through their coat protein (HAstVs-CP) that interacts with both C1q and MBL, resulting in the inhibition of both the CP and LP (71).

Another mechanism adopted by some pathogens to interfere with the complement system’s initial steps is the acquisition of regulators involved in this stage, such as C1-INH. For instance, *E. coli* O157:H7 produces the secreted protease of C1 esterase inhibitor (StcE), which cleaves C1-INH and potentiates its inhibitory effect, thus downregulating the activation of both the CP and LP (44). *B. recurrentis*, the causative agent of louse-borne relapsing fever, is able to acquire the complement regulator C1-INH by the surface protein complement inhibition (CihC) (41). Likewise, *Bordetella pertussis*, binds the same regulator by the auto transporter protein virulence-associated gene 8 (Vag8) (42). The merozoites of the malaria causing parasite *Plasmodium falciparum* adopt that same strategy via its *P. falciparum* merozoite surface protein-3 (PfMSP3.1) (66).

#### 1.3.2 Interfering with the intermediate stages of the complement cascade: a decisive battle

**Figure 3:**
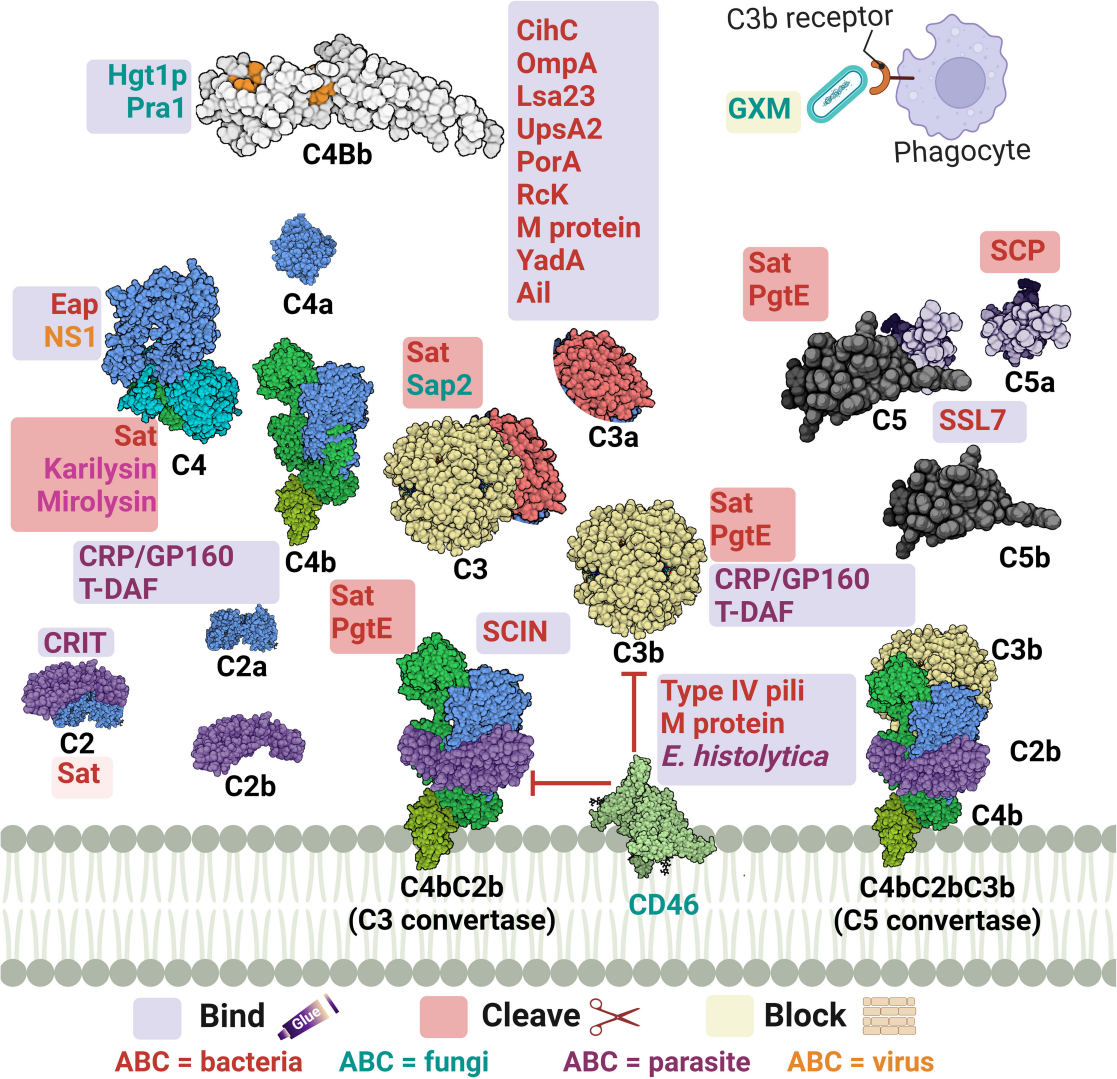
A schematic diagram showing microbial virulence factors involved in targeting this part of the complement cascade. The labels for the bacterial factors are shown in red, the fungal ones in turquoise, the parasite-related ones in purple, and finally the viral factors in orange. The mechanism by which those proteins affect the pathway is categorized into binding, shaded in purple; cleavage, which is shaded in dark pink; and blocking, which is highlighted in beige. The proteins’ codes and structures were retrieved from the Protein Data Bank, while the figure was generated using BioRender.

**Figure 4:**
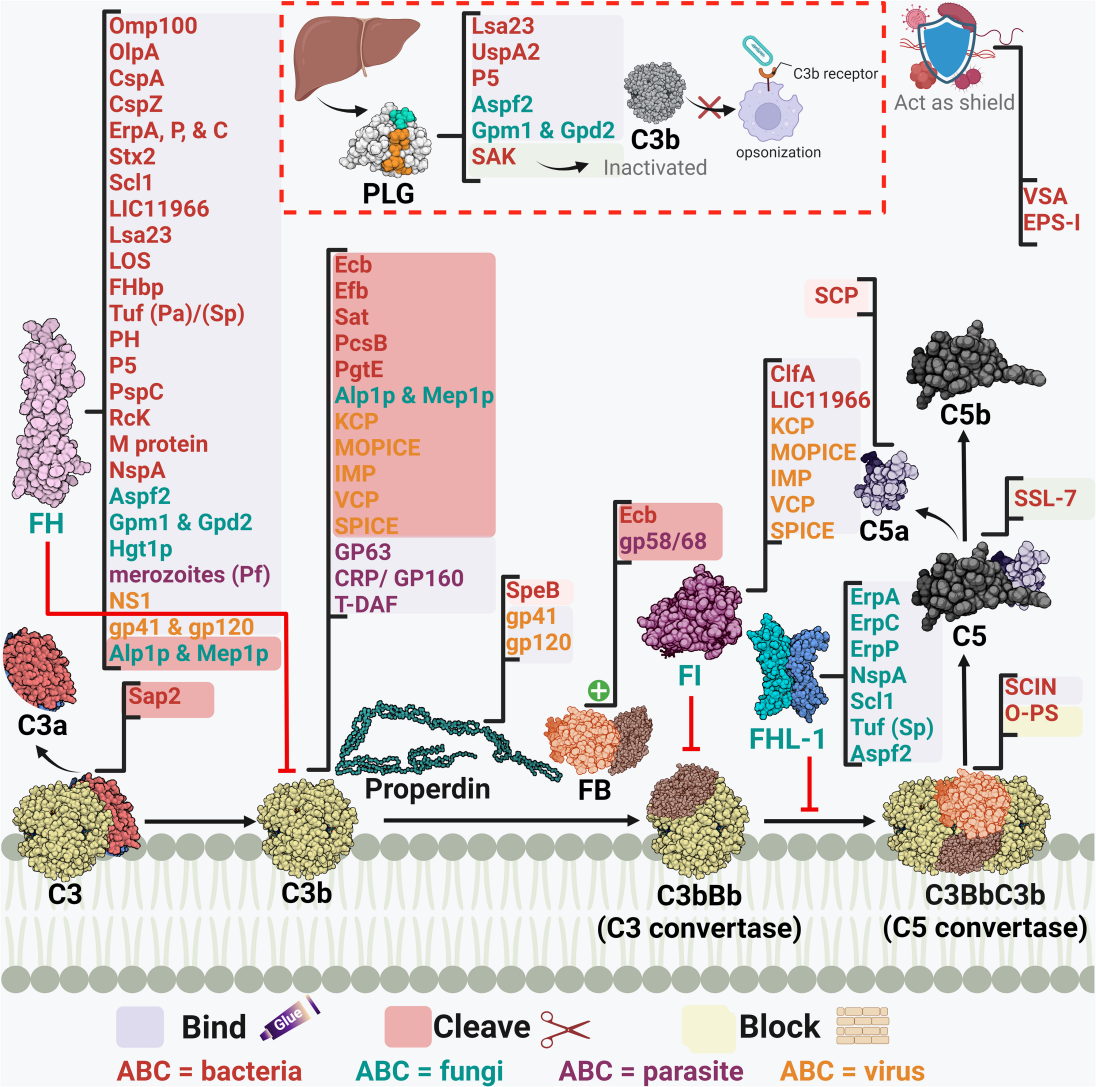
A schematic diagram showing microbial virulence factors involved in targeting this part of the complement cascade. The labels for the bacterial factors are shown in red, the fungal ones in turquoise, the parasite-related ones in purple, and finally the viral factors in orange. The mechanism by which those proteins affect the pathway is categorized into binding, shaded in purple; cleavage, which is shaded in dark pink; and blocking, which is highlighted in beige. The proteins’ codes and structures were retrieved from the Protein Data Bank, while the figure was generated using BioRender.

Following complement initiation, the intermediate stage of the cascade aims at the subsequent activation of the complement components through two convertases: the C3 and C5 convertases. The C3 convertase is generated as a common product for the first step of complement activation in all three pathways. However, the composition of the C3 convertase is C4bC2b in both CP and LP, while for the AP, it is C3bBb (37). This intermediate stage of the cascade is a choke point in complement evasion not solely because the three networks compile to generate those two functionally similar components, but also, as C3 cleavage gives way to two essential effector fragments, C3a and C3b (72). The mechanisms deployed by microbial pathogens to interfere with this stage are summarized in **Table 2** and **Figures 3**, **4**.

**Table 2:** Microbial strategies for interfering with the complement cascade intermediate stages.

Organism	Microbial factor	Action	Ref
A) Bacteria			
*A. actinomycetemcomitans*	Omp100	Binds FH	(73)
*Borrelia* spp.			
i) *B. burgdorferi*	CspA, CspZ, ErpA, P, & C	Bind FH and FHL-1	(74–76)
ii) *B. recurrentis*	CihC	Binds C4BP	(41)
*B. pertussis*	BrkA	Blocks complement deposition strating from C4	(77)
*B. pseudomallei*	O-PS	Prevents C3 convertase formation close to the cell surface	(78)
*E. coli*	OmpA	Binds C4BP	(79)
	Stx2 (EHEC)	Binds FH, FHR-1, and FHL-1	(80)
	Sat	Degrades C2, C3, C3b, C4, C4b, & C5	(81)
*H. influenzae*			
i) Encapsulated	PH	Binds FH	(82)
ii) NTHi	P5	Binds FH & PLG	(83)
*L. interrogans*	Lsa23	Binds C4BP, FH, & PLG	(84)
*Leptospira* spp.	LIC11966	Binds FH & FI	(85)
*M. catarrhalis*	UspA2	Binds C4BP & PLG	(86, 87)
	OlpA	Binds FH	(88)
*M. pulmonis*	VSA & EPS-I	Act as sheild	(89)
*Neisseria* spp.	LOS	Binds FH	(90)
	PorA	Binds C4BP	(91)
	FHbp	Binds FH	(92)
	NspA	Binds FH & FHL-1	(93)
	Type IV pili	Binds MCP (CD46)	(94)
*P. aeruginosa*	Tuf	Binds FH	(95)
*Salmonella enterica*	RcK	Binds FH & C4Bp	(96, 97)
	PgtE	Cleaves C3b, C4b and C5	(98)
*S. aureus*	ClfA	Binds FI	(99)
	Eap	Binds C4	(100)
	Ecb & Efb	Binds C3b. Bind C3d-containing molecules	(101, 102)
	SAK	Activates PLG	(51)
	SCIN	Binds C3 convertases & blocks their activation	(103)
	SSL-7	Binds C5	(104)
*Streptococcus* spp.			
i) GAS/GBS, C & G streptococci	SCP	Inactivates C5a	(105)
ii) *S. mitis*	PcsB	Decreases C3b/iC3b deposition	(106)
iii) *S. pyogenes*	Scl1	Recruits FHR-1 and FH.	(107)
	SpeB	Degrades properdin	(108)
	M protein	Binds FH, FHL-1, C4BP, & CD46	29 (109)
iv) *S. pneumoniae*	PspC & Tuf	bind FH	(110–112)
*T. forsythia*	Karilysin & Mirolysin	Degrades C4	(59, 60)
*Yersinia* spp.			
Y. enterocolitica	YadA	Binds C4BP	(113)
*Y. pestis*	Ail	Binds C4BP	(114)
B) Fungi			
*A. fumigatus*	Aspf2	FH, FHL-FHR-1, & PLG	(115, 116)
	Alp1p & Mep1p	Supports FI-mediated cleavage of C3b into iC3b.	(117)
*C.albicans*	Gpm1 & Gpd2	bind FH, FHL-1, & PLG	(118, 119)
	Hgt1p	Binds FH & C4BP	(120)
	Pra1	Binds C4BP	(121)
	Sap2	Degrades C3	(122, 123)
*C. neoformans*	GXM	Blocks complement receptors on phagocytes	(124)
C) Viruses			
Flaviviruses			
i) WNV	NS1	Binds FH Binds C4 & C1s ➔depletes C4 in solution	(125, 126)
ii) DENV			
iii) YFV			
HIV-1	gp41 & gp120	Bind FH & properdin	(127)
KSHV	KCP	DAF for C3 convertases & degrades C3b (cofactor for FI)	(128)
Poxviruses			
i) Monkeypox	MOPICE	Degrades C3b (cofactor for FI)	(129)
ii) Cowpox	IMP	DAF for C3 convertases & degrades C3b (cofactor for FI)	(130)
iii) Vaccinia virus	VCP		(131, 132)
iv) Variola virus	SPICE		(133)
D) Parasites			
*E. histolytica*		Acquires MCP & DAF via trogocytosis	(134)
*L. major*	GP63	Inactivates C3b to iC3b	(135)
*P. falciparum*	merozoites	Bind FH & FHL-1	(136)
*T. cruzi*	CRIT	Binds C2	(137)
	CRP/GP160	Binds to C3b and C4b ➔ dissociates the C3 convertases	(138)
	gp58/68	Binds FB ➔ blocks AP C3 convertase	(139)
	T-DAF	Binds C3b and C4b ➔ blocking C3 convertase	(136)

The acquisition of the hosts’ regulators is a very prominent strategy in this step as the labyrinth of multiple regulators and co-factors represents a possible getaway for microorganisms. Both C4BP and FH are complement regulators that bind to host cell surfaces, along with other FI co-factors, and are considered high-value targets for pathogens at this point (32).

*Actinobacillus actinomycetemcomitans*, which is associated with periodontal diseases, uses its outer membrane protein 100 (Omp100) to deposit FH at the cell surface and evade the complement (73). *Neisseria* spp. express lipooligosaccharides (LOS), similar to the host glycans, that enables them to recruit host regulators. Also, the sialylation of these LOS increases FH binding (90). Furthermore, the surface-exposed proteins porin A (PorA) and factor H-binding protein (FHbp) bind both C4BP (91) and FH (92), respectively enabling them to control the three pathways. Moreover, the binding of MCP (CD46) has been also demonstrated by its type IV pili (94). Similarly, *P. aeruginosa* binds FH by its translation elongation factor Tu (Tuf) (95). The encapsulated *H. influenzae* uses its protein H (PH) to bind FH while the Nontypeable *H. influenzae* (NTHi) uses its protein 5 (P5) to bind to the same regulator (82, 171). P5 was also shown to bind to PLG and cleave C3b following activation (83). *Salmonella enterica* serovars Typhimurium and Enteritidis harbour a virulence plasmid encoding the RcK outer membrane protein that recruits FH to inactivate the C3b (96). The same protein was also shown to interfere with the CP and LP via binding to C4BP (97).

*Borreliaburgdorferi* has several proteins on its surface that bind FH and FHL-1 such as complement regulator-acquiring surface protein (CRASP-1 or CspA) (76), complement regulator-acquiring surface protein 2 (CRASP-2 or CspZ) (75), and OspEF-related protein (Erp) types A, P, and C (74). The CihC protein of *B. recurrentis* that interferes with the complement cascade initiation plays another role in this stage by binding to the C4BP regulator (41).

As for *S. pneumoniae*, both its surface protein C (PspC) and elongation factor Tu (Tuf) can bind to FH (110–112). Moreover, the streptococcal M protein is capable of binding to 4 regulators: FH, FHL-1, C4BP, and CD46 (29, 109). Similarly, *Leptospira interrogans* uses its leptospiral surface adhesions 23 (Lsa23) to bind to multiple regulators including C4BP for the CP, FH for the AP, and PLG to cleave the C3b (84). In addition, the Shiga toxin 2 (Stx2) of the enterohemorrhagic *E. coli* (EHEC) binds to FH, FHR-1, and FHL-1 (80). The neisserial surface protein A (NspA) was shown to bind to both FH & FHL-1; however, the meningococcal NspA displayed an enhanced binding ability to these regulators over the *N. gonorrhoeae* homolog (93).

The binding of C4BP is seen in *B. pertussis* via its filamentous hemagglutinin (FHA) surface protein and another unidentified moiety; however, *fha* mutants are still as sensitive to killing by the complement system as their wild-type counterparts, suggesting that FHA could be playing a negligent role in the complement resistance of this pathogen (172). Moreover, C4BP is bound by multiple other pathogens, including *Yersinia pestis* by the attachment invasive locus (Ail) (114), *Y. enterocolitica* by the yersinia adhesin A (YadA) (113), *E. coli* by the outer membrane protein A (OmpA) (79), and *Moraxella catarrhalis* by the ubiquitous surface proteins A2 (UspA2) and to a lesser extent A1 (UspA1) of some *M. catarrhalis* strains (86). UspA2 derived from other serum-resistant *M. catarrhalis* strains was found to be unable to bind the C4BP regulator, suggesting that this is not the only role of UspA2 in evading killing by the complement system (153). Interestingly, the same surface protein was shown to bind PLG resulting in the degradation of both C3b and C5 (87). Also, it has been shown that the Opa-like protein A (OlpA) in some *M. catarrhalis* strains can bind FH and hence interferes with the AP (88). Moreover, *S. aureus* binds the AP regulator FI using its clumping factor A (ClfA). This binding promotes the breakdown of the surface-bound C3b into iC3b (99). Last but not least, *Leptospira* spp. express the LIC11966, which is an ErpY-like lipoprotein that can bind FH and FI (85).

Fungal evasion of the complement system at this stage can also happen through the recruitment of host regulators. This is seen in *C. albicans* which binds C4BP with its pH-regulated antigen 1 (Pra1) (121). On the other hand, both the surface protein phosphoglycerate mutase 1 (Gpm1) and the glycerol-3-phosphate dehydrogenase 2 (Gpd2) bind FH, FHL-1, and PLG (118, 119), while the high affinity glucose transporter 1 (Hgt1p) binds FH and C4BP (120). Additionally, *C. albicans* interferes with complement activation and complement mediated opsonization through its secreted aspartic proteases (Sap1-3), which degrade both C3b and C4b (123). *Aspergillus fumigatus* conidia avoids recognition by the complement system through the acquisition of some host regulators such as FH, FHL-FHR-1, and PLG with the help of its Aspf2 protein (115, 116). It is worth mentioning that other developmental stages of this fungi bind to these regulators with lower capacity, if any.

Moving to the viral side, in HIV, the acquisition of the host regulators occurs through the interaction between the host FH and properdin and the viral envelope proteins gp41 and gp120 (127). Also, the flaviviruses non-structural protein 1 (NS1) present in West Nile virus (WNV), dengue virus (DENV), and yellow fever viruses (YFV) binds FH which leads to blocking the AP (125, 126).

Another example, but this time from the perspective of parasites, is the larval stage of the cestode *Echinococcus granulosus*, which releases an FH binding molecule from the wall of the hydatid cyst that limits AP amplification (173, 174). Moreover, when *P. falciparum* merozoites are exposed to human serum they recruit FH and FHL-1 acting as a complement downregulation strategy (136). *Entamoeba histolytica* develops resistance to the complement by trogocytosis, a smart way of acquiring multiple membrane bound complement host regulators, that might be working collectively, via ingesting small bites of the host cells (134). Among those regulators are the MCP and DAF which are regulators of the intermediate stage of the complement cascade, in addition to CD59 which is a regulator of the late stages as will be discussed later.

Away from binding to or recruiting the host’s regulators, pathogens have adopted other approaches to interfere with the steps leading to C3 convertase formation or activation. The *S. aureus* pathogen possesses an arsenal of evasion proteins that target the intermediate stages of the complement cascade. Some strains use the extracellular adherence protein (Eap) to bind C4b and block the assembly of the C3 convertase, hence, targeting both the LP and CP (100). Other strains use the SAK protein which functions as a PLG activator to cleave bound C3b and block opsonization (51). They also use the *Staphylococcus* complement inhibitor (SCIN) which inhibits both C3 convertases by binding and stabilizing them and preventing the addition of more C3b to either of them (103). *S. aureus* is capable of blocking the activation and amplification loop by binding the staphylococcal extracellular fibrinogen binding protein (Efb) and its homologous extracellular complement binding protein (Ecb) to C3b and hence preventing the binding of Factor B to C3b and thus the AP C3 convertase is not formed (101, 102). The *Streptococcus* spp. are an equally strong foe, as the group A *S. pyogenes* (GAS) produce the streptococcal pyrogenic exotoxin B (SpeB) which degrades the AP regulator properdin (108). It was also reported that the overexpression of the surface immunodominant protein PcsB of *S. mitis* resulted in the reduction of the C3b/iC3b that are deposited on the cell surface (106). The auto-transporter of *B. pertussis* named Bordetella resistance to killing A (BrkA) is reported to reduce complement component deposition starting from C4, interfering with CP and LP, and moving all the way to MAC formation (77). The karilysin and mirolysin of *T. forsythia* exhibit efficient degradation capacity for C4 which blocks both CP and LP (59, 60). *Salmonella enterica* uses its outer membrane aspartate protease PgtE to cleave C3b, C4b, and C5 and it also activates the PLG, which in return increases its complement resistance. (98). Recently, the *E. coli* secreted autotransporter toxin (Sat) was shown to degrade multiple complement components involved in the three pathways including C2, C3, C3b, C4, C4b, and C5 (81).

In the case of fungi, some of them adopt the approach of inhibiting the cleavage of key complement proteins which in return inhibits opsonization and phagocytosis. For instance, *C. albicans* has multiple surface proteins, such as the *C. albicans* phosphoglycerate mutase1 (Gpm1p), that bind host PLG (118) and inhibit the cleavage of C3, C3b, and C5, and in return inhibits the generation of both C3b and C5 (112). *C. albicans* also secretes aspartic protease (Sap2) which cleaves C3 (122). On another front, other fungi adopt a different approach to degrading the key complement components, such as *A. fumigatus* which uses its secreted alkaline proteases (Alp1p) from the hyphal morphotype and the metalloprotease (Mep1p) from the conidial morphotype to deploy this approach. FH is cleaved by Mep1p without losing its cofactor activity. Then, FH supports FI-mediated cleavage of C3b into the inactivated form (iC3b). Thus, the fungal pathogen can be easily disseminated in the invaded host (117). A third approach, used by *Cryptococcus neoformans*, is by blocking the complement receptors on phagocytes and hence interfering with complement-dependent phagocytosis, which is done by the production of glucuronoxylomannan (GXM) (124).

Viruses, on the other hand, have versatile strategies for inhibiting the intermediate stages in the complement cascade. The Kaposi’s sarcoma-associated human herpes virus (KSHV) complement control protein (KCP) regulates the complement cascade by its DAF activity, which aids in accelerating the C3 convertases’ decay. At the same time, it provides cofactor activity for FI which leads to the degradation of both C4b and C3b (128). On the other hand, the NS1 protein of flaviviruses was shown to bind both C4 and C1s. This binding enhances the cleavage of C4 to C4b in solution and depletes the C4 supply, resulting in the blockage of both CP and LP (126). Poxviruses are known for their ability to evade the complement system via several proteins, the first of which to be discovered was the vaccinia virus complement control protein (VCP). Initially, studies showed that VCP binds C3b and C4b and possesses a decay accelerating activity (131). Then, its cofactor activity for FI was highlighted (132). Orthologs of the VCP are present in other poxviruses, such as the smallpox inhibitor of complement enzymes (SPICE) in the variola virus and the monkeypox inhibitor of complement enzymes (MOPICE). Those VCP orthologs are capable of inhibiting C3b and C4b with different potencies (133, 175). Yet the MOPICE functions only through its cofactor activity and lacks decay acceleration (129). Also, the cowpox virus VCP homolog, termed as the inflammation modulatory protein (IMP), possesses cofactor activity and binds C3b and C4b to inhibit the classical and alternative pathways (130).

As for parasites, they have their own tricks when it comes to inhibiting the C3 convertase. For example, *T. cruzi* employs surface molecules, such as complement C2 receptor inhibitor trispanning (CRIT), which binds C2 and prevents its cleavage by C1s or MASP2– blocking C3 convertase formation (137). Moreover, *T. cruzi* expresses the complement regulatory protein (CRP/GP160) that binds to C3b and C4b and dissociates the C3 convertases (138). In addition, the same parasite can specifically interfere with the AP C3 convertase using its glycoprotein “gp58/68” that inhibits the formation of cell-bound and fluid-phase alternative pathway C3 convertase through binding to FB (139). Also, the T-DAF binds C3b and C4b which blocks C3 convertase formation (176). On the other hand, *Leishmania* mainly uses the surface protease gp63 to inactivate C3b and convert it to iC3b (135).

*Mycoplasma pulmonis* does not interfere with the complement activation; however, it evades the complement and the phagocytosis process using a shielding strategy. The variable surface antigens (VSA) and the exopolysaccharide I (EPS-I) both contribute to shielding the pathogen against killing by the complement system and potentially other harmful molecules (89). Also, the elongated O-antigenic polysaccharide (O-PS) moieties of *Burkholderia pseudomallei* stretches away from the cell surface, a strategy that prevents complement-mediated killing by blocking C3 convertase formation close to the cell surface, and subsequently the membrane insertion of the TCC (78).

Efb-C and its homolog Ecb are both indispensable for the *S. aureus* fight against the complement system through their previously mentioned ability to prevent AP C3 convertase formation. Moreover, they possess the ability to bind C3d-containing molecules (C3, C3b, iC3b, and C3d) and they can impede all C3b-containing convertases which comprise the C5 convertases of all three complement pathways (102). Factor H-related protein-1 (FHR-1) is one of five other factor H-related (FHR) proteins that were initially identified to have complement inhibitory action by competing with FH and potentially FHL proteins. Organisms were found to recruit those proteins for their own benefit, such as special serotypes of *S. pyogenes* (M6 and M55) which recruit FHR-1 as well as FH by the streptococcal collagen-like protein 1 (Scl1) (107).

Stabilization of the AP C5 convertase by properdin is imperative for the cascade progression, as it is considered the main supplier of C5b to the terminal pathway (11). Stemming from this fact comes the smart strategy used by GAS to disrupt C5 convertase assembly by the cleavage of properdin through the SpeB (108). Contrary to properdin, DAF accelerates the convertases’ decay, a mechanism that is used by *T. cruzi* through its DAF-mimicking glycoprotein T-DAF (136).

Among the staphylococcal superantigen-like (SSL) proteins is SSL-7 which binds C5 and accordingly hinders its binding to the C5 convertase and prevents the production of C5a. Through this mechanism, *S. aureus* could evade complement killing by phagocytosis. Although SSL-7 was shown to form a complex with C5b and prevent MAC formation, this is unrelated to *S. aureus* complement evasion ability as it is already resistant to killing by MAC (104). Streptococci, including groups A, B, C, and G of human origin, produces a C5a peptidase (SCP) which specifically degrades and inactivates C5a. This complement component is considered a major human phagocyte chemotaxin. Accordingly, the SCP action delays the influx of phagocytes and aids in the pathogenesis of these pathogens (105).

Orthopoxviruses, including variola homologs, secrete soluble viral complement regulatory proteins, such as the smallpox inhibitor of complement enzymes (SPICE), which inhibit the formation of the C3/C5 convertases necessary for viral clearance (133).

#### 1.3.3 Interference with the terminal stages: the final round

**Figure 5:**
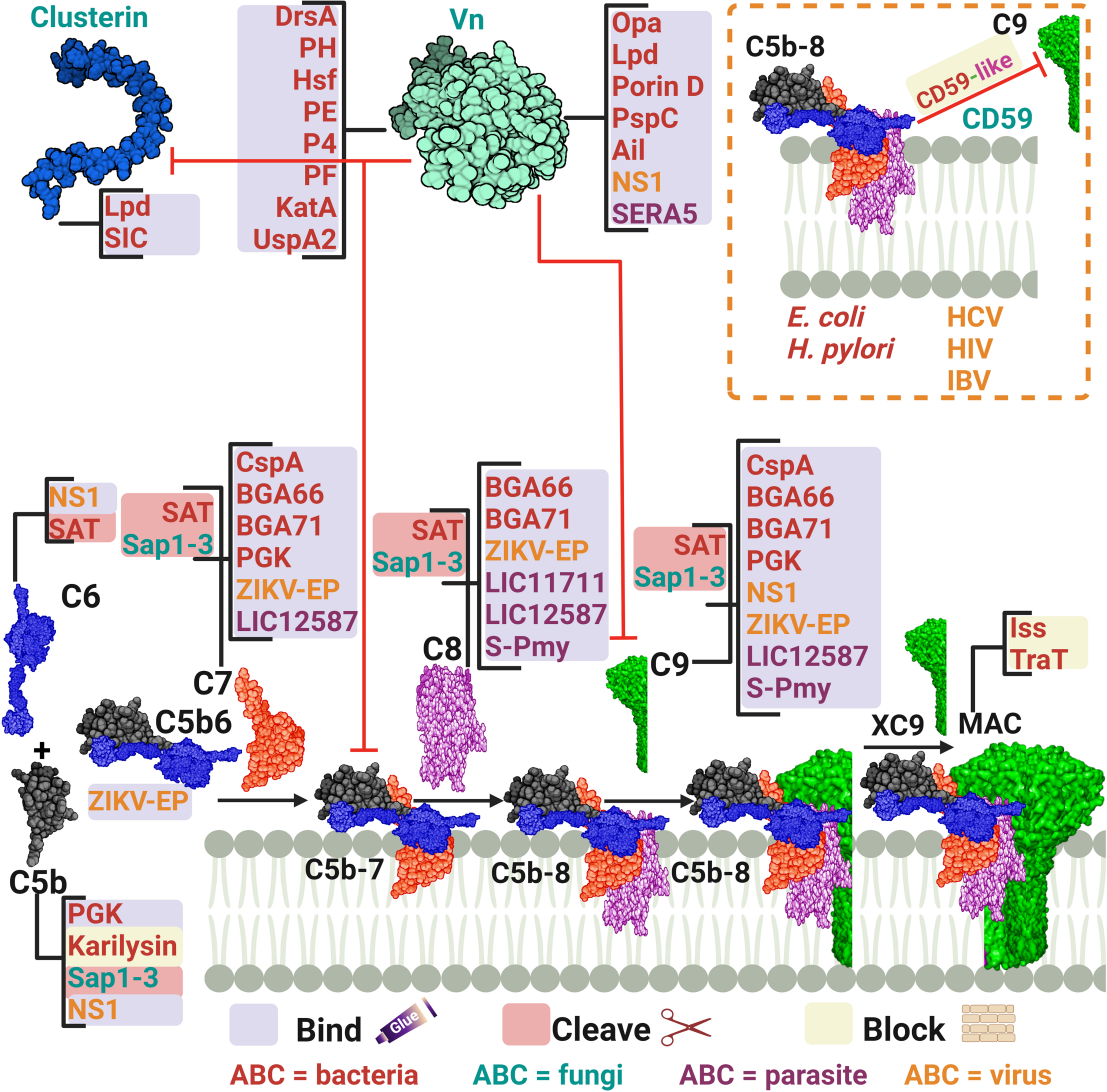
A schematic diagram showing microbial virulence factors involved in targeting the final stage of the complement cascade. The labels for the bacterial factors are shown in red, the fungal ones in turquoise, the parasite-related ones in purple, and finally the viral factors in orange. The mechanism by which those proteins affect the pathway is categorized into binding, shaded in purple; cleavage, which is shaded in dark pink; and blocking, which is highlighted in beige. The proteins’ codes and structures were retrieved from the Protein Data Bank, while the figure was generated using BioRender.

As the complement system goes through its terminal step, the microbial counter defences work to counteract the deleterious effects of the terminal complement complex (TCC) (**Figure 5**; **Table 3**). A successful microbial evasion strategy is the acquisition of the host complement regulators to block the harmful effects of this immune system component. TCC formation is regulated by multiple host regulators that microbes have already exploited to evade the lethal effect of MAC formation.

**Table 3:** Microbial strategies for interfering with the complement cascade late stages.

Organism	Microbial factor	Action	Ref
A) Bacteria			
*Borrelia* spp.			
i) *B. afzelii*	CspA	Binds C7 & C9	(140)
ii) *B. bavariensis*	BGA66 & BGA71	Binds C7, C8 & C9	(141)
iii) *B. burgdorferi*	CspA	Binds C7 & C9	(142)
iv) *B. spielmanii*	CspA	Binds C7 & C9	(140)
*E. coli*	Iss & TraT	Blocks TCC function	(143)
		Incorporates host CD59	(144)
	Sat	Degrades C6, C7, C8 & C9	(81)
*H. ducreyi*	DsrA	Binds to Vn	(145)
*H. influenzae*			
i) Encapsulated	PH	Binds to Vn	(146)
ii) Type b	Hsf	Binds to Vn	(147)
ii) NTHi	PE	Binds to Vn	(148)
	P4	Binds to Vn	(149)
	PF	Binds to Vn	(150)
*H. pylori*	KatA	Binds to Vn	(151)
		Incorporates host CD59	(152)
*M. catarrhalis*	UspA2	Binds to Vn	(153)
*Neisseria* spp.			
*N. meningitidis*	Opa	Binds to Vn	(154)
*P. aeruginosa*	Lpd	Binds Vn & clusterin	(155)
	Porin D	Binds to Vn	(156)
*Streptococcus* spp.			
*S. pyogenes*	SIC	Binds clusterin and blocks the uptake of C567 onto cell membranes	(157)
*S. pneumoniae*	PGK	Binds C5, C7 & C9	(158)
	PspC	Binds Vn	(159)
*T. forsythia*	Karilysin	Inhibit C5 deposition	(67)
*Y. pestis*	Ail	Binds to Vn	(160)
B) Fungi			
*C.albicans*	Sap1-3	Degrades C5 and late components	(123)
C) Viruses			
Flaviviruses			
i) WNV	NS1	Binds Vn, C5, C6, & C9	(161)
ii) DENV	NS1		
iii) ZIKV	NS1		
	E protein	Binds C5b6, C7, C8, & C9	(162)
HCV	HCV-CP	Inhibits C9 promoter activity	(163)
		Incorporates host CD59	(164)
HIV		Incorporates host CD59	(165)
IBV		Incorporates host CD59	(166)
D) Parasites			
*L. interrogans*	LIC11711	Binds C8 & PLG	(167)
	LIC12587	Binds C7, C8, & C9	(167)
*N. fowleri*	CD59-like	Binds C9	(168)
*P. falciparum*	SERA5	Binds Vn	(169)
*Schistosoma* spp.	S-Pmy	Binds C8 & C9	(170)

Many pathogens can bind vitronectin (Vn). They either use bound-Vn to prevent complement lysis or to “hitchhike” their way into cells. UspA2, the previously discussed protein of *M. catarrhalis*, was found to render complement resistance to some strains by binding Vn and hindering C9 polymerization and thus preventing MAC assembly (153). This was confirmed by testing *uspA2* mutant strains which showed serum-sensitivity and by testing the wild-type strain in a Vn-depleted serum which led to a bactericidal action. *Haemophilus ducreyi* secretes a UspA2 homologue protein termed *H. ducreyi* serum resistance protein A (DsrA) which was confirmed to have a Vn binding ability (145). Likewise, *H. influenzae* expresses its pervasive adhesion protein E (PE) which binds Vn and hence inhibits the MAC formation, and increases the bacterial resistance among both non-typable and encapsulated *H. influenzae* (148). In the case of *H. influenzae* serotype f, protein H (PH) was shown to bind Vn to evade killing by human serum and also to better adhere to alveolar epithelial cells (146). *H. influenzae* serotype b, the most virulent of the encapsulated strains, utilizes its Haemophilus surface fibrils (Hsf) auto-transporter to bind both soluble and immobilized forms of Vn and enhance its serum survival (147). Moreover, two additional proteins of the non-typeable *H. influenzae* (NTHi) were shown to utilize the same tactic of recruiting Vn: protein 4 (P4) and protein F (PF) (149, 150). Another outer membrane adhesion protein is the opacity-associated outer membrane protein (Opa), already proven to confer resistance upon *N. meningitidis*, was found to bind active human Vn and hence, promoting cellular invasion (154). In *P. aeruginosa*, two proteins were reported to bind Vn, dihydrolipoamide dehydrogenase (Lpd) and Porin D. Lpd, a moonlighting protein offering multifunctional physiological activities independent of each other, was able to bind Vn to varying degrees among the tested strains (155). While porin D, an outer membrane protein, was shown to bind Vn via a proteomic approach (156). Both proteins effectively bound Vn and subverted TCC formation in bacterial clinical isolates. Moreover, *Helicobacter pylori* uses the moon-lighting approach to the phenomenon of additional, unrelated functions in often highly conserved proteins display the hydrogen peroxide-neutralizing enzyme catalase (KatA) on its surface and binds Vn to subvert the complement system (151). Also, the *Y. pestis* Ail protein was shown to bind functional Vn to evade TCC attack (160).

Vn acquisition is not an exclusive approach used by bacteria only as it is also found in viruses. The NS1 of the Dengue virus together with that of WNV and Zika virus (ZIKV), binds Vn directly and forms an NS1-Vn complex that inhibits C9 polymerization. This complex was identified in infected patients’ plasma, although data did not correlate its presence to disease progression (161). The serine repeat antigen 5 (SERA5) of the *P. falciparum* was also shown to bind Vn through its P47 N-terminal domain (169).

Gram-positive bacteria do not necessarily need to deploy evasion tactics against MAC formation owing to their possession of a hypertrophic peptidoglycan layer that shields the bacterial plasma membrane against this lytic attack (24, 26). Nevertheless, they do exploit a number of evasion strategies against MAC, something that could be explained by the recent structural analysis of the MAC which suggests activities beyond membrane penetration (24). Additionally, findings state that the whole C5b-9 is deposited on Gram-positive bacteria, yet the functional role is still not clear (177). In line with this, *S. pneumoniae* employs its multifunctional choline-binding protein PspC to hinder the terminal pathway by binding Vn. This was backed up by the lower levels of vitronectin binding detected in *pspC* mutants deficient in this protein (159).

Besides Vn, clusterin was found to be another pivotal key regulator of the TCC. *P. aeruginosa* Lpd was shown to bind this protein, besides binding to Vn, resulting in a reduction in C5b-9 deposition (155). For Gram-positive bacteria, the streptococcal inhibitor of complement (SIC) is an abundant protein secreted by *S. pyogenes* that shows comparable efficiency in binding to clusterin, although the biological relevance of this binding is doubtful (157). At the same time, it was shown that SIC can behave as the fluid phase regulator clusterin in blocking the uptake of the C5b-7 complex onto cell membranes, yet how this could benefit *S. pyogenes* remains debatable (157).

CD59, also known as protectin, is a glycophosphoinsitol (GPI) membrane bound human regulator of the complement that protects host cells from MAC-mediated lysis. Pathogens have acquired a tactic to incorporate the released GPI from the host cells into their membrane to protect themselves from TCC formation. For instance, it was shown that CD59 was functionally active after being inserted, in a Ca^2+^ dependent mechanism, in the membrane of two non-encapsulated deep rough *E. coli* strains (144). Moreover, *H. pylori* CagA-positive strains successfully incorporated the host CD59 and this binding was inversely proportional to the amount of deposited C5b-9 on the cell surface (152). Among viruses, HCV has demonstrated a similar capability of incorporating CD59 in its envelop (164). Also, the infectious bronchitis virus (IBV) adopted a similar tactic to evade the antibody-dependent complement-mediated lysis (166). In addition, HIV acquires protectin from the host cell surface to evade killing and ensure infectivity (165).

Other pathogens that do not incorporate the regulator CD59 have developed the smart tactic of expressing CD59-like proteins. For instance, the amebae *Naegleria fowleri*, which is responsible for primary amoebic meningoencephalitis, possess an anti-CD59 monoclonal antibody-reactive surface protein that was immunoprecipitated with C9 from human serum. At the same time, the complement sensitive non-pathogenic *N. gruberi* amebae lacked these reactions (168). Schistosomes have been identified to possess CD59-like protein homologs, yet their role in complement evasion remains unclear (178).

Another tactic used by microbes to interfere with the late stages of complement activation is to bind the components of the TCC, making them unavailable for membrane insertion and cell lysis. For example, the CspA of *B. burgdorferi, B. afzelii*, and *B. spielmanii*, can bind complement proteins C7 and C9, simultaneously, thus inhibiting TCC-mediated cellular destruction (140, 142). *B. bavariensis* has two surface proteins, termed BGA66 and BGA71, that contribute to its serum resistance by directly binding to C7, C8, and C9 and hence, preventing MAC formation (141). BGA71 can only inhibit TCC at the C7 level, while BGA66 interferes with TCC formation at different steps (141). Phosphoglycerate kinase (PGK), a pneumococcal glycolytic enzyme previously identified as a PG-binding protein of *S. pneumoniae*, was imputed to have an additional role as a MAC inhibitor and was found to simultaneously bind complement proteins C5, C7, and C9 (158). The Sat of *E. coli*, in addition to the earlier components mentioned above, are capable of degrading C6, C7, C8, and C9 (81). The karilysin of *T. forsythia* can inhibit C5 deposition, allowing enhanced serum survival and limiting MAC assembly (67). Moreover, the *C. albicans* (Sap1-3) proteases described above also degrade C5 and complement late components (123).

In viruses, the NS1 of the Dengue virus binds to complement proteins C5, C6, and C9 (161). While the Zika virus E protein binds C5b6, C7, C8, and C9 (162). Instead of depleting the TCC components, HCV suppresses the TCC by modulating complement protein synthesis through its viral core protein, which inhibits C9 promoter activity (163). *L. interrogans* has two surface exposed proteins, LIC11711 and LIC12587, which bind different components of the TCC, interfering with the MAC formation. LIC11711 was found to bind C8 in addition to PLG, while LIC12587 was able to bind C7, C8, and C9 (167). Finally, the paramyosin of *S. mansoni*, a multi-helical charged protein, can bind C1, C8, and C9, utilizing its highly charged zones (170).

*E. coli* strains harboring the R100 plasmid and the ColV/BM plasmid can express the outer membrane protein TraT and the increased serum survival (Iss) protein, respectively (143). Both proteins conferred a significant increase in *E. coli* survival in the presence of serum. However, the consumption of C6, C7, C8, and C9 was not altered when compared to non-expressing cells, indicating that it is the function of the TCC that is blocked, not its formation (143).

## 2 Discussion

The complement system was discovered more than 130 years ago; however, it was not until 70 years later that we realized microbes are able to evade this system with diverse strategies that have evolved over time. According to the survey conducted in this study, bacteria account for the majority of the microbial components researched, while fungal factors are the least commonly studied ones. However, in comparison with bacterial factors, viral and parasitic factors are still less studied.

Looking closer at the bacterial factors, multiple bacterial species can interfere with the complement system at the three stages of the complement cascade: early, intermediate, and late. For example, *Borrelia* spp., *E. coli*, *Neisseria* spp., *P. aeruginosa*, *Streptococcus* spp., and *T. forsythia* all have multiple virulence factors that can target the complement cascade, from blocking activation to interfering with terminal stages. *S. aureus* interferes with only the first two stages of the complement system; however, it has more than 10 virulence factors at its disposal, preventing the complex complement system from removing it while inside the mammalian host.

Examining the pathogens and their complement evasion tools, the spectrum of virulence factors is diverse. For instance, *T. forsythia* uses karilysin to interfere with the three stages of complement activation and *Schistosoma* spp use S-Pmy to block both activation and late stages of the complement system. On the other hand, *S. aureus*, although it targets only the first two stages, uses different virulence factors for each stage. Similarly, *C. albicans* uses seven virulence factors to target the intermediate and late stages.

When we compared the three complement stages in terms of the number of microbial factors involved, the intermediate stage was the most targeted with more than 40 reported virulence factors. This could possibly be attributed to the multiple complement components involved in this stage, especially the convertases, as well as the regulators that span across the three complement pathways. Interestingly, factor H is at the top of the list of the most targeted complement components with at least 28 virulence factors derived from the four main types of pathogens that interact with it to interfere with its role in AP activation.

It is noteworthy that most of the studies reported here have demonstrated, via various approaches, that binding of the pathogen to a complement component or regulator results in a functional role in complement evasion. For instance, this was demonstrated by generating isogenic mutants in the gene(s) encoding the proposed virulence factor and showing that this mutant is more sensitive to complement-mediated killing as compared to their wild-type (55, 56, 121, 159). More provided evidence was that the depletion of the serum from the complement regulator results in increasing susceptibility of the resistant wild-type strain to complement-mediated killing (153). Moreover, the silencing of the virulence factor or immunizing the host against it results also in an increased susceptibility to the complement (40). In addition, the proposed mechanism was supported by the ectopic expression of the virulence factor in a sensitive strain which rendered it resistant to killing by the complement system (41).

On the other hand, in some situations, proposed bacterial virulence factors exhibited good binding to the complement regulators or components, yet this was not reflected in a functional phenotype. For instance, the binding of the C4BP to *B. pertussis* FHA (172). Also, although the Mac/IdeS of the Group A *Streptococus* was shown to specifically cleave IgG interfering with the CP activation, yet knocking out the gene encoding this protease had no notable impact on multiple GAS immune evasion phenotypes (179).

Understanding the host-pathogen interactions during complement evasion is a corner stone in the treatment of several human diseases. Mimicking or inhibiting the microbial factors produced by bacteria, viruses, fungi, and parasites provides a potential platform as novel classes of antimicrobials and complement-targeting therapeutics.

## 3 Conclusion

The complement system plays a pivotal role in the innate immune system’s battle against invading pathogens; however, pathogens fight back in constantly evolving ways to evade this system. In this article, we have assessed more than 180 research papers to present a comprehensive overview of different mechanisms used by microorganisms to evade the complement system. We classified the pathogens’ interference with the system into three main phases: influencing the complement activation, interfering with the intermediate stages of the complement cascade, and finally, interfering with the terminal stages of membrane attack complex formation. For pathogens to achieve their goal of influencing the complement system they can mask their surface molecules, secrete proteins that inhibit or degrade complement components, recruit complement regulatory proteins, or modify their own structures to become more resistant. At each phase of the complement cascade, pathogens can deploy multiple evasion mechanisms, and use different strategies to counteract the complement system on multiple levels. Understanding how the complement system is controlled allows us to better comprehend the possible mechanisms that pathogens use to evade it. Moreover, keeping track of how pathogens are evolving in terms of their evasion strategies is crucial for developing new strategies to combat them.

## Author contributions

MH: Formal analysis, Investigation, Methodology, Writing – original draft, Data curation, Visualization. HN: Data curation, Formal analysis, Writing – review & editing. DM: Data curation, Writing – original draft. NA: Data curation, Writing – original draft. AA: Writing – original draft, Conceptualization, Formal analysis, Funding acquisition, Investigation, Methodology, Project administration, Resources, Writing – review & editing.
